# O^6^-Methylguanine-DNA Methyltransferase (MGMT): Challenges and New Opportunities in Glioma Chemotherapy

**DOI:** 10.3389/fonc.2019.01547

**Published:** 2020-01-17

**Authors:** Wei Yu, Lili Zhang, Qichun Wei, Anwen Shao

**Affiliations:** ^1^Department of Radiation Oncology, The Second Affiliated Hospital, Zhejiang University School of Medicine, Hangzhou, China; ^2^Cancer Institute (Ministry of Education Key Laboratory of Cancer Prevention and Intervention), Zhejiang University Cancer Institute, Hangzhou, China; ^3^Department of Neurosurgery, The Second Affiliated Hospital, Zhejiang University School of Medicine, Hangzhou, China

**Keywords:** MGMT, methylation, alkylating agents, target therapy, chemotherapy

## Abstract

Chemoresistance has been a significant problem affecting the efficacy of drugs targeting tumors for decades. MGMT, known as O^6^-methylguanine-DNA methyltransferase, is a DNA repair enzyme that plays an important role in chemoresistance to alkylating agents. Hence, MGMT is considered a promising target for tumor treatment. Several methods are employed to detect MGMT, each with its own advantages and disadvantages. Some of the detection methods are; immunohistochemistry, methylation-specific PCR (MSP), pyrophosphate sequencing, MGMT activity test, and real-time quantitative PCR. Methylation of MGMT promoter is a key predictor of whether alkylating agents can effectively control glioma cells. The prognostic value of MGMT in glioma is currently being explored. The expression of MGMT gene mainly depends on epigenetic modification–methylation of CpG island of MGMT promoter. CpG island covers a length of 762 bp, with 98 CpG sites located at the 5' end of the gene, ranging from 480 to 1,480 nucleotides. The methylation sites and frequencies of CpG islands vary in MGMT-deficient tumor cell lines, xenografts of glioblastoma and *in situ* glioblastoma. Methylation in some regions of promoter CpG islands is particularly associated with gene expression. The change in the methylation status of the MGMT promoter after chemotherapy, radiotherapy or both is not completely understood, and results from previous studies have been controversial. Several studies have revealed that chemotherapy may enhance MGMT expression in gliomas. This could be through gene induction or selection of high MGMT-expressing cells during chemotherapy. Selective survival of glioma cells with high MGMT expression during alkylating agent therapy may change MGMT status in case of recurrence. Several strategies have been pursued to improve the anti-tumor effects of temozolomide. These include the synthesis of analogs of O^6^-meG such as O^6^-benzylguanine (O^6^-BG) and O^6^-(4-bromothenyl) guanine (O^6^-BTG), RNAi, and viral proteins. This review describes the regulation of MGMT expression and its role in chemotherapy, especially in glioma. Targeting MGMT seems to be a promising approach to overcome chemoresistance. Further studies exploring new agents targeting MGMT with better curative effect and less toxicity are advocated. We anticipate that these developments will improve the current poor prognosis of glioma patients.

## Introduction

**Figure 1:**
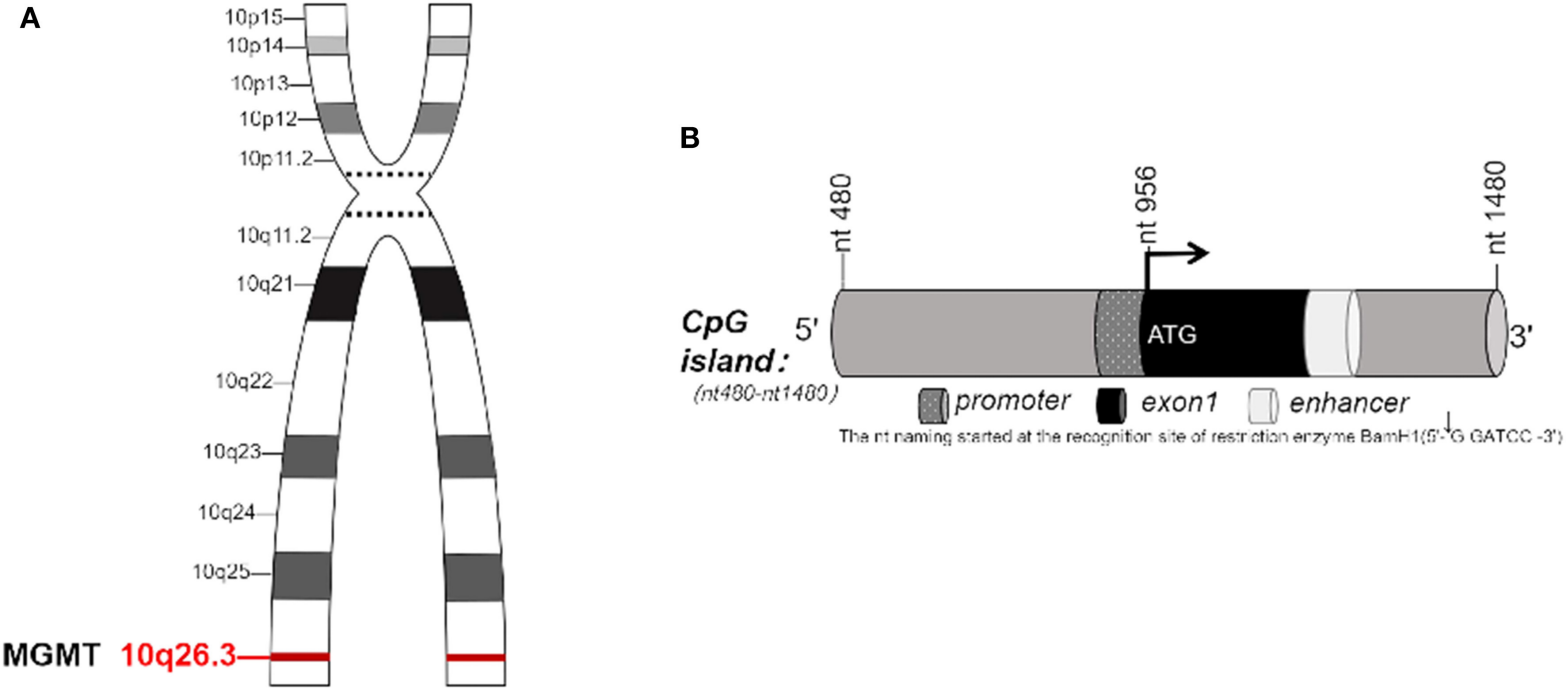
**(A)** MGMT gene is located on chromosome 10, q26.3. **(B)** CpG island in MGMT. **(B)** In 1987, Gardiner-Garden and Frommer (1) identified the CpG island with 762 bp in MGMT gene. It has 98 CpG sites, located on about 480–1,480 nucleotide (nt) at the 5' end of this gene. The transcription start site (TSS) is located at nt956, CpG island covers a length of 500 bp at both 5' end and 3' end of TSS. The name of nt was first coined by Harris et al. (2), derived from the recognition site of restriction enzyme BamH1.

O^6^-methylguanine-DNA methyltransferase, known as MGMT, is a DNA “suicide” repair enzyme. It repairs damaged guanine nucleotides by transferring the methyl at O^6^ site of guanine to its cysteine residues, thus avoiding gene mutation, cell death and tumorigenesis caused by alkylating agents. MGMT gene is located on chromosome 10q26.3 (Figure 1A), with a total length of 300,437 bp (3, 4). The expression of MGMT gene is mainly regulated by epigenetic modification. Many studies have shown that the loss of MGMT expression is not due to gene deletion, mutation, rearrangement or unstable RNA, but due to methylation of CpG island of MGMT promoter (5–9). In 1987, Gardiner and Frommer discovered that the human MGMT gene has a CpG island with a length of 762 bp, with 98 CpG sites located at the 5' end of the gene, ranging from 480 to 1,480 nucleotides (nt) (Figure 1B). The transcription initiation site of the gene is nt 956, and the CpG island spans about 500 bp (1) at the 5' and 3' ends of the transcription initiation site. The nt naming was initiated by Harris et al. beginning from the recognition site (2) of the restriction enzyme BamH1. In the non-methylated state, the transcriptional initiation sites of MGMT adhere to four precisely located nucleosome-like structures, which fine-tunes the transcription of the gene. Methylation of CpG islands leads to heterochromatinization, accompanied by rearrangement and random localization of nucleosomes, thus obscuring the transcription initiation sites and making transcription devices unable to bind (10, 11). Other studies have shown that methylation and chromatin status modulate the transcription of MGMT gene by determining whether Sp1 and other transcription factors access the MGMT promoter (12).

## Detection Methods for MGMT

Immunohistochemistry is a semi-quantitative method used to detect the expression of MGMT protein (13–15). It can distinguish between tumor cells and non-tumor cells, and thus the results are not confounded by the heterogeneity of tumors, but this method is greatly influenced by the subjectivity of the observer (16). In clinical research, many methods are used to detect the methylation status of MGMT promoter in glioma. The most frequently used method is methylation-specific PCR (MSP) (17, 18). This method was first established in 1996 for detection of promoter methylation (19). However, it can only detect the methylation of small fragments complementary to primers, and cannot determine the exact location of promoter methylation. Therefore, it is a semi-quantitative method (20). Whether conventional MSP primers (+120 toc +143, +173 to +196) bind to the key regions that regulate MGMT expression remains unclear. Pyrophosphate sequencing is a comprehensive sequencing method. In this method, methylation level of a single CpG site modified by sulfite can be quantitatively evaluated by efficient PCR amplification and sequencing. Therefore, Pyrophosphate sequencing is more reliable than MSP (21). However, the role of CpG island hypermethylation in gene silencing remains controversial (22–25). Many researchers hold the view that methylation of MGMT promoter directly inhibits gene transcription, thus detection of promoter methylation may serve as an indicator of susceptibility to alkylating agents. In fact, the most direct method used to detect the sensitivity of alkylating agents is MGMT activity test. The number of MGMT active molecules per unit protein or DNA detected by MGMT activity test can reflect the level of MGMT protein and RNA. However, because the test requires fresh or frozen tissues and *in situ* hybridization, it is not feasible for daily application. In contrast, quantitative detection of MGMT RNA expression by real-time quantitative PCR seems more suitable and highly sensitive, but few studies have applied it in MGMT RNA detection. Recently, Wang et al. found that a combination of immunohistochemistry and qMSP assays can provide high sensitivity and specificity for the prediction of MGMT status (26).

## The Prediction and Prognostic Value of MGMT Promoter Status in Glioma

The MGMT gene encodes a DNA damage repair protein that removes alkylating agents resulting in resistance to chemotherapy. Because DNA methylation can inhibit transcription, methylation of MGMT promoter increases sensitivity to alkylating agents (27). Several studies have shown that methylation of MGMT promoter can predict whether alkylating agents can be of benefit in glioblastoma and low-grade gliomas (28–37). Two other clinical trials have revealed that methylation status of MGMT promoter can predict the prognosis of glioma patients. In these two studies, retrospective analysis of MGMT promoter methylation in elderly patients found that it could predict good prognosis in temozolomide (TMZ) group, but not in radiotherapy alone group (38, 39). The EORTC26951 clinical trial retrospectively analyzed the methylation status of MGMT promoter in anaplastic oligodendroglioma patients. It was found that methylation of MGMT promoter in anaplastic oligodendroglioma patients predicted better overall survival (OS) and PFS, whether in radiotherapy alone or in sequential radiotherapy and chemotherapy group [chemotherapy regimen: procarbacine, lomustine (CCNU), vincristine (PCV)]. But it had no prognostic value in glioblastoma patients. Elsewhere, it has been reported that methylation of MGMT promoter has no predictive value for chemosensitivity of anaplastic oligodendroglioma patients undergoing adjuvant PCV chemotherapy (40). Another phase III randomized clinical trial, NOA-04, drew a similar conclusion that methylation of MGMT promoter and IDH1 mutation reduces the risk of progression in anaplastic glioma patients, and patients with MGMT promoter methylation have a longer PFS (41) in both radiotherapy and chemotherapy groups (PVC). In addition, results from a phase III clinical trial prospectively indicate that MGMT promoter methylation status can be used as a biomarker to predict good prognosis of glioblastoma patients treated with TMZ (42) (Table 1).

**Table 1:** Summary of the OS and PFS of patients receiving different treatments and characterized by non-methylated and methylated MGMT promoters in different studies.

**References**	**Pathology**	**Treatment**	**OS (months)**			**PFS (months)**		
			**MGMTm**	**MGMTu**	**All**	**MGMTm**	**MGMTu**	**ALL**
Criniere et al. (28)	GBM	RT+BCNU	17.1 (14.5–26.5)	13.1 (10.1–17.2)	NG	NG		
		RT	15.1 (9.8–n.r.)	10.2 (3.33–21.9)	NG			
		ALL 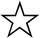	14.4 (13–16.1)	13.6 (11.4–15.7)	13.9 (12.5–15.3)	7.33 (5.8–8.43)	7.63 (6.47–8.63)	7.37 (6.5–8.43)
Hegi et al. (29)	GBM	RT+TMZ	21.7 (17.4–30.4)	12.7 (11.6–14.4)	NG	10.3 (6.5–14.0)	5.3 (5.0–7.6)	NG
		RT	15.3 (13.0–20.9)	11.8 (9.7–14.1)		5.9 (5.3–7.7)	4.4 (3.1–6.0)	
		ALL	18.2 (15.5–22.0)	12.2 (11.4–13.5)		NG	NG	
Reifenberger et al. (30)	GBM (age ≥ 70)	RT+TMZ	13.1 (11.0–15.3)	10.4 (8.4–12.4)	12.3(11.2–13.4)	7.3 (6.2–8.5)	7.2 (5.6–8.7)	7.2 (6.3–8.0)
		TMZ	7.2 (5.6–8.9)	2.6 (n.r.)	6.8(4.8–8.8)	6.8 (2.5–11.0)	0.5 (n.r.)	5.3 (0.1–10.5)
		RT	7.8 (3.4–12.2)	8.8 (7.5–10.1)	8.7(7.0–10.4)	4.5 (3.5–5.4)	5.2 (4.3–6.2)	5.0 (4.4–5.6)
		No treatment	2.3 (0.8–3.8)	2.0 (0.6–3.7)	2.3(0.9–3.7)	1.8 (1.1–2.4)	1.7 (0.4–3.1)	1.8 (1.0–2.5)
		ALL	8.4 (6.7–10.1)	6.4 (3.9–8.9)	7.7(6.3–9.0)	5.2 (4.3–6.1)	4.7 (3.8–5.5)	4.8 (4.3–5.3)
Esteller et al. (31)	AA/GBM	ALL (RT+BCNU)	MGMTu/MGMTm: HR = 9.5 (95% CI: 3.0–42.7, *p* < 0.001)			MGMTu/MGMTm: HR = 10.8 (95% CI: 4.4–30.8, *p* < 0.001)		
Hegi et al. (32)	GBM	ALL (RT+TMZ after surgery)	MGMTu/MGMTm: The risk of death within 18 months after surgery: 92% vs. 38%; *p* = 0.002			NG		
Everhard et al. (33)	LGG	ALL (TMZ)	NG			29.5 (21.5–n.r.)	6 (5–n.r.)	28 (20–n.r.)
Pandith et al. (37)	Gliomas	RT+TMZ	40.1 (29.8–50.3)	6.8 (3.8–9.6)	43.4 (32.5–54.1)	23.9 (20.0–27.7)	3.2 (0.6–5.8)	25.8 (21.9–29.6)
Malmstrom et al. (38)	GBM (age≥60)	TMZ	9.7 (8.0–11.4)	6.8 (5.9–7.7)	8.3 (7.1–9.5)	NG		
		Standard RT (60 Gy)	8.2 (6.6–9.9)※	7.0 (5.7–8.3)※	6.0 (5.1–6.8)			
		Hypofractioned RT (34 Gy)			7.5 (6.5–8.6)			
		ALL	9.0 (8.0–10.0)	6.9 (5.9–7.9)	NG			
Wick et al. (39)	AA/GBM (age ≥ 65)	TMZ	n.r. (10.1–n.r.)	7 (5.7–8.7)	8.6 (7.3–110.2)	8.4 (5.5–11.7)	3.3 (3.0–3.5)	3.3 (3.2–4.1)
		RT	9.6 (6.4–n.r.)	10.4 (8–11.6)	9.6 (8.2–10.8)	4.6 (4.2–5.0)	4.6 (3.7–6.3)	4.7 (4.2–5.2)
		ALL	11.9 (9.0–n.r.)	8.2 (7.0–10.0)	NG	5.7 (5.0–7.4)	3.5 (3.3–3.7)	NG
van den Bent et al. (40)	AOD/AOA (≥25% oligodendroglia elements)	RT RT+PVC	59.3 (30.0–66.2) n.r. (n.r.)	12.3 (11.5–28.5) 19.0 (12.3–34.5)	NG	17.9 (11.9–43.4) 49.0 (19.1–71.2)	7.8 (7.1–17.6) 10.5 (5.2–23.0)	NG
Wick et al. (41)	Anaplastic gliomas (WHO III)		NG			MGMTu/MGMTm:		
		RT			72.1 (n.r.)	HR = 2.0 (95% CI: 1.1–3.6, *p* < 0.03)		30.6 (16.3–42.8)
		TMZ/PCV			82.6 (n.r.)	HR = 2.7 (95% CI: 1.4–5.1, *p* < 0.003)		31.9 (21.1–37.3)
Gilbert et al. (42)	GBM	Standard dose TMZ	21.4 (17.6–29.0)	14.6 (13.2–16.5)	16.6 (14.9–18.0)	6.5 (4.1–9.6)	5.1 (4.3–5.7)	5.5 (4.7–6.1)
		Dense dose TMZ	20.2 (15.4–25.1)	13.3 (12.3–14.3)	14.9 (13.7–16.5)	10.1 (7.9–12.4)	6.0 (5.5–6.5)	6.7 (6.2–7.7)
		ALL	21.2 (17.9–24.8)	14.0 (12.9–14.7)	NG	8.7 (6.6–11.2)	5.7 (5.1–6.1)	NG

*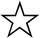, included other treatments: BCNU alone or supportive care; ※, standard RT and hypofractioned RT is grouped together; GBM, glioblastoma; LGG, low-grade gliomas; AA, anaplastic astrocytoma; AOD, anaplastic oligodendroglia tumors; AOA, anaplastic oligoastrocytoma; RT, radiotherapy; TMZ, temozolomide; BCNU, carmustine; PCV, procarbazine, lomustine, and vincristine; OS, median overall survival; PFS, median progression-free survival; MGMTm, MGMT promoter methylated status; MGMTu, MGMT promoter unmethylated status; NG, not given; n.r., not reached; HR, hazard ratio; 95%CI, 95 percent confidence interval*.

## Epigenetic Regulation of MGMT CpG Island

The methylation sites and frequencies of CpG islands vary among MGMT-deficient tumor cell lines, xenografts of glioblastoma and *in situ* glioblastoma. Pieper et al. used Linker-mediated PCR (LMPCR) to detect the methylation status of MGMT promoter. It was found that the changes in methylation level of MGMT promoter mainly occurred at four CpG loci in cell lines expressing MGMT and those lacking MGMT, rather than being distributed uniformly throughout the CpG island. Two of them are located at about 130 nucleotides (+130) downstream of the transcription initiation site (TSS), including the sites recently studied using MPS. Two other nucleotides (−200) are located upstream of the transcription initiation site, and the transcription factor binding sites in both cell lines are not methylated. The transcription initiation site is defined as 0 (43). Watts et al. performed bisulfite sequencing PCR (BSP) on 108 CpG loci of 8226/s and 8226/v promoter CpG islands, respectively, and found that 8226/v has three methylation-rich regions which differs from those of 8226/s: −446 to −353, −265 to −162 and +112 to +212 (11). Costello et al. analyzed the methylation status of CpG loci in MGMT promoter −252 to −155 and −90 to +65 regions of glioma cell line by LM-PCR, and found that 21 of 25 loci were negatively correlated with MGMT gene expression (12). Because the authors detected the methylation level in high, low and non-expressing cell lines in these two regions, a quantitative relationship could not be established. Qian et al. used BSP method to detect the methylation level of CpG loci from −249 to +259 in MGMT CpG island region. It was found that HT29, a cell line expressing MGMT, was almost not methylated in this region, whereas BE, a cell line not expressing MGMT, was heavily methylated in each clone in this region. The most frequently methylated regions ranged from −249 to −103, +107 to +196 (44). Malley et al. used pyrophosphatic acid sequencing to detect the methylation of CpG islands in the entire MGMT promoter of glioblastoma cell lines, xenografts and normal brain tissues (41 samples). It was found that the +152 to +214 were the key regions promoting the transcription of MGMT (45). Subsequently, methylation of MGMT promoter was studied in human glioma samples. It was found that the methylation of CpG loci at −186 to −172 and +93 to +153 regions was most correlated with MGMT gene expression, but previous MSP loci were not found in this region, although the methylation level of MSP loci was similar to that reported previously (46). Bady et al. used human methylation 450 gene chip (HM-450K) to detect 14 CpG loci of MGMT promoter in 63 glioblastoma samples. It was found that the methylation of −193 and +173 CpG loci was negatively correlated with gene expression and had a good predictive accuracy for prognosis (47). Similarly, Mur et al. obtained genome-wide methylation profiles of 247 glioma samples from HM-450K platform, including 25 CpG loci in CpG island of MGMT promoter region. The methylation of + 173 CpG loci was significantly associated with overall survival (48). These researchers also found that MGMT promoter CpG islands are not suitable for methylation and this do not regulate expression or predict the prognosis of patients. Everhard et al. found that MGMT promoter regions −452 to −399 were highly methylated in both tumors and normal brain tissues. The region −90 to +69 is the first CpG region of small promoter, TSS and non-coding exon, which is equivalent to the methylation-free region (46) in both normal brain tissue and tumors. Thus, transcriptional silencing does not require methylation of the entire CpG island, but only methylation of several gene-specific core CpG sites. Therefore, methylation at some regions of promoter CpG islands is particularly associated with gene expression.

## The Influence of Chemotherapy and Radiotherapy on the Methylation Status, Activity and Protein Expression of MGMT

Methylation of the promoter region of the MGMT gene is known to predict the response to alkylating agent's treatment in glioma patients. However, knowledge about the change in the methylation status of the MGMT promoter after chemotherapy, radiotherapy or both is still incomplete. Wiewrodt et al. analyzed MGMT activity in 40 paired primary and recurrent glioblastomas, 16 patients after RT only, 24 patients with RT combined with chemotherapy (TMZ and/or CCNU or ACNU). In both recurrent groups, the MGMT activity was higher than in primary tumors. In contrast, for patients who received RT only, there was no significant difference between primary glioblastomas and recurrences. The MGMT activity was significant, however, in patients with primary glioblastomas and recurrences that received RT plus alkylating agent therapy (49). Brandes et al. analyzed MGMT promoter methylation status of 38 paired primary and recurrent glioblastomas treated with adjuvant radiotherapy and chemotherapy. They found that MGMT methylation status was changed in 14 patients (37%) who had recurrent tumors and more frequently in those with methylated MGMT than in unmethylated patients (50). Christmann et al. compared MGMT activity and MGMT promoter methylation in 46 primary glioblastoma samples and 19 recurrent glioblastoma samples. They found that MGMT activity increased after treatment, and methylation of MGMT promoter was detected in 39% primary tumors, while only 5.3% recurrent glioblastomas displayed MGMT promoter methylation (17). Elsewhere, Felsberg et al. analyzed the methylation status of MGMT promoter in 80 paired primary and recurrent glioblastomas, of which 16 patients received radiotherapy alone and 64 patients received radiotherapy and TMZ chemotherapy. They found the MGMT methylation status of 89% patients was not altered (51). It is worth noting, besides, that the response of the human MGMT promoter to genotoxic stress may be weak. Although Fritz et al. and Chan et al. had reported MGMT mRNA transcription can be induced by DNA-damaging treatments, both of their experiments were limited in rat H4IIE hepatoma cells (52, 53). As for human MGMT promoter, Grombacher et al. found that it could be induced by dexamethasone when transfected into rat H4IIE and human HeLa S3 (Mex+) cells, but methylating agents and ionizing radiation only worked in H4IIE cells (54). Boldogh et al. analyzed the mechanism of human MGMT expression induction, they found that protein kinase C-mediated signaling played an important role, involving activation of AP-1 sites on MGMT promoter by TPA (55). Aasland et al. further identified that human MGMT promoter can be induced by glucocorticoids, but not by genotoxic stress, in human malignant glioblastoma cells (56). They put forward that a cluster of SP1 sites in human MGMT promoter prevented transcriptional up-graduation and overshadowed activation signals from other weaker transcriptional factors. The transcription factor SP1 was sequestrated by p53, which was induced following radiochemotherapy (57). Coincidentally, an earlier retrospective clinical data from Pitter at al. showed longer survival of no glucocorticoid usage GBM patient cohorts, alongside corresponding data in animal models (58). Thus, radiation and chemotherapy may have minor influence on transient transcriptional activation of human MGMT. The finding of a protection of tumor cells by dexamethasone and other steroids suggests that a controlled use of glucocoricoids in GBM therapy is desirable. In conclusion, these studies revealed that chemotherapy may provoke an up-regulation of MGMT expression in gliomas through selection of high MGMT expressing cells during chemotherapy. Selective survival of glioma cells with high MGMT expression during alkylating agent therapy may change MGMT status when recurrence.

## MGMT in Glioblastoma Stem Cells

Cancer stem cells have been implicated in the progression and recurrence of GBMs. It has been recognized that even after effective treatment of tumors, minimal residual stem cells may be activated to enter a new stage of differentiation and proliferation. In this way, cancer stem cells promote the recurrence of tumors. Thus, we postulate that glioblastoma stem cells may cause resistance to TMZ, which enables them survive during chemotherapy. Liu et al. and Pistollato et al. revealed that glioblastoma (GBM) stem cells, identified with the stem cell marker CD133, express high level of MGMT and displayed strong tumor resistance to TMZ (59, 60). Beier et al. reported that there are distinct stem cell populations that, despite having similar MGMT promoter methylation status, differ in MGMT protein expression. And they also found that TMZ preferentially kills cancer stem cells in glioblastoma in MGMT-negative cell lines (61, 62). Mantwill et al. stated that MGMT is not expressed in all stem cell lines, which indicates that these cells have different grades of TMZ resistance (63). Happold et al. observed that differentiation of glioma stem cells resulted in a gradual loss of MGMT expression and increased TMZ sensitivity (64, 65). Although MGMT is highly expressed in stem cells, it is not clear why the alkylating agents are not effective in recurrent GBMs. Do the differentiated cells retain the TMZ resistance features of stem cells? These challenges necessitate the search for the mechanisms that regulate the expression of MGMT in different cell stages.

## Targeting MGMT Protein

**Figure 2:**
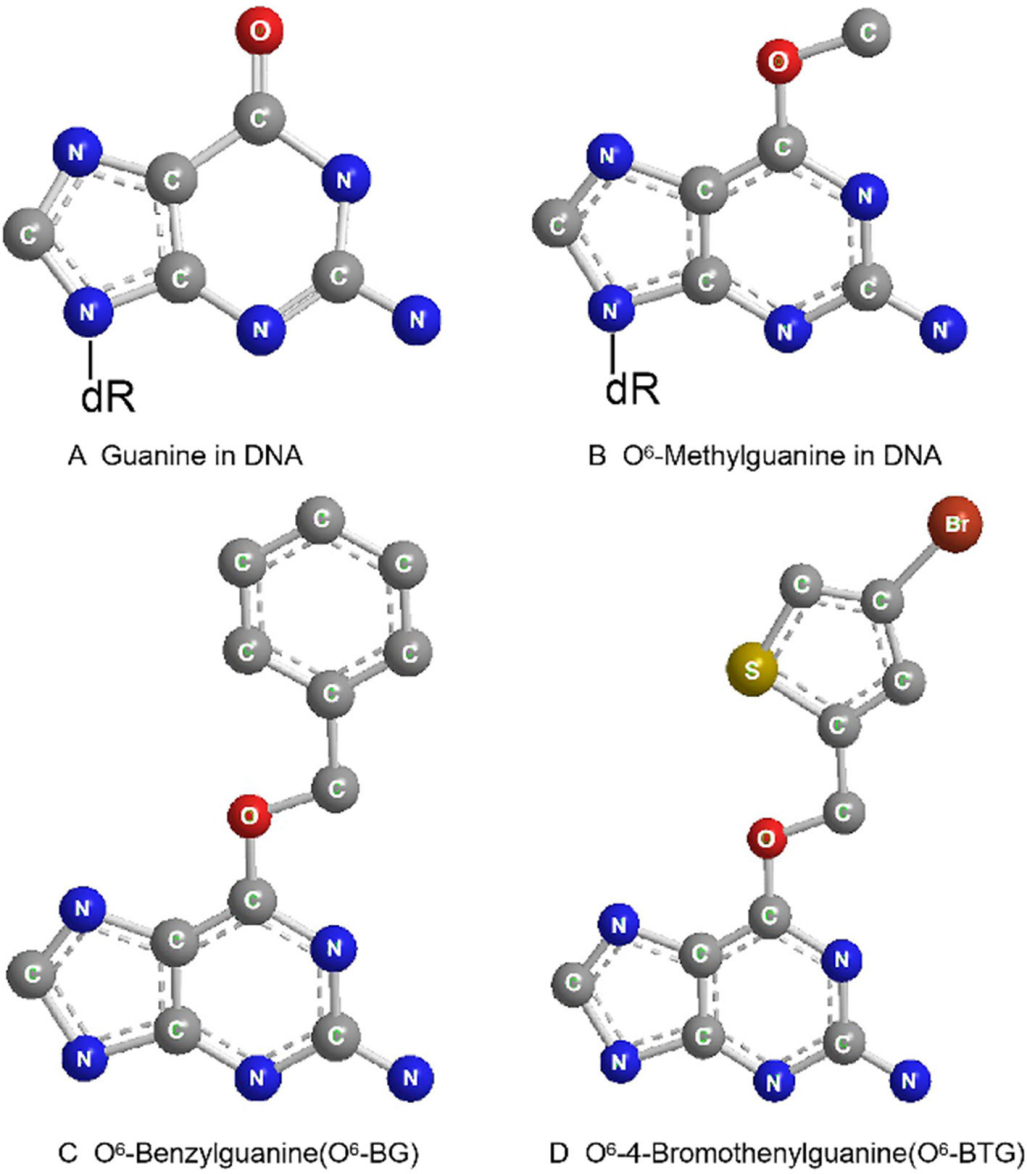
Molecular structure of guanine in DNA **(A)**, O^6^-Methylguanine in DNA **(B)**, O^6^-Benzylguanine **(C)**, and O^6^-4-Bromothenylguanine **(D)**.

O^6^-benzylguanine (O^6^-BG) is the analog of O^6^-meG which is a low molecular weight pseudosubstrate for MGMT. It inactivates MGMT through alkyl group transfer (Figure 2). It can pass the blood brain barrier and has, therefore, the potential to be a treatment for gliomas. It has been widely used as an MGMT inhibitor and as a sensitizer of glioma cells to alkylating agent TMZ (66, 67). Koch et al. found that local intracranial interaction of O^6^-BG with TMZ after intraoperative removal of brain tumors might delay tumor recurrence without any side effect (68). Phase I, II and III clinical trials of O^6^-BG combined with TMZ have revealed that this combination successfully aberrates other tumors, such as brain tumor, melanoma, lymphoma and colon cancer (69–72). A later phase II clinical research by Quinn et al. found that 06-BG combined with gliadel wafer prolonged the survival time of patients. However, it also increased the risk of hydrocephalus, cerebrospinal fluid (CSF) leak, and CSF/brain infection (73).

Another pseudosubstrate, O^6^-(4-bromothenyl) guanine (O^6^-BTG), has 10-fold higher potency than O^6^-BG in inactivating MGMT protein and is orally bioavailable without inherently toxic (Figure 2). It has been reported that O^6^-BTG efficiently and rapidly inactivates MGMT in various tumors *in vivo* and *in* v *vitro* and significantly increases tumor sensitivity to TMZ (74–77). A phase I trial of O^6^-BTG in combination with TMZ in advanced solid tumors established an oral ATase-depleting dose of lomeguatrib (Trade name for O6-BTG) and developed a combination regimen with TMZ that was 75% of the maximum tolerable dose of the single agent. The dose-limiting toxicity of O^6^-BTG was myelosuppression (78). Papachristodoulou et al. reported that a liposomal O^6^BTG can efficiently target MGMT, thereby sensitizing murine and human glioma cells to TMZ *in vitro* and magnetic resonance image-guided microbubble-enhanced low-intensity pulsed focused ultrasound mediates the delivery of the stable liposomal MGMT inactivator into the tumor region resulting in complete MGMT depletion *in vivo* (79).

Although the developed MGMT inhibitors, O^6^-BG and O^6^-BTG, are effective, their systemic toxicity due to non-specific targeting to normal cells cannot be ignored. Going forward, the high glucose consumption hallmark of tumor cells presents a new avenue that can be exploited for development of selective inhibitors by conjugating agents to glucose. It has been reported that both O^6^BG-Glu and O^6^BTG-Glu are highly effective at inhibiting MGMT in several cancer cell lines, including T98G glioblastoma. These agents also enhance the cell-killing effect of temozolomide (80–82). Besides, Tomaszowski et al. found that glucose conjugates are subject of transport out of the cell by the ATP-binding cassette (ABC) transporter mediated efflux, which impacts the efficiency of MGMT inhibition. In this study, the importance of proper linker selection for a successful ligand-based drug delivery strategy was underscored (83). Similarly, conjugating pseudosubstrates to folate esters is another promising strategy to target tumor cells (84). So far, few studies have investigated the cellular effects of glucose or folate esters conjugated inhibitors. Further detailed studies should unravel the mechanisms of these inhibitors to provide better treatment agents.

Watson et al. conducted phase II clinical trials to evaluate the efficacy of lomeguatrib in patients with melanoma. They found that lomeguatrib plus TMZ had a greater MGMT inactivation than did TMZ treatment alone (85). Another study by the same group also reported that lomeguatrib can be applied in prostate, primary CNS, and colorectal cancers to inactivate MGMT (86). However, lomeguatrib increases myelosuppression, and other studies revealed that it did not improve the response rate to TMZ (69, 70, 74, 87–89).

RNA interference is another promising therapy targeting MGMT. Kato et al. reported that when combined with TMZ, the MGMT-siRNA/liposome complex exerted a strong synergistic antitumor effect (90). Zhang et al. found that miR-181d downregulated MGMT by directly interacting with MGMT 3'UTR, and this potentiated the TMZ sensitizer as an MGMT targeting therapy (91). Nie et al. found that miR-198 directly targeted MGMT by binding to the 3′-UTR of MGMT, thereby inhibiting the MGMT mRNA translation in GBM cells. MiR-198 restored the tumor sensitivity to TMZ in glioblastomas overexpressing MGMT (92).

**Figure 3:**
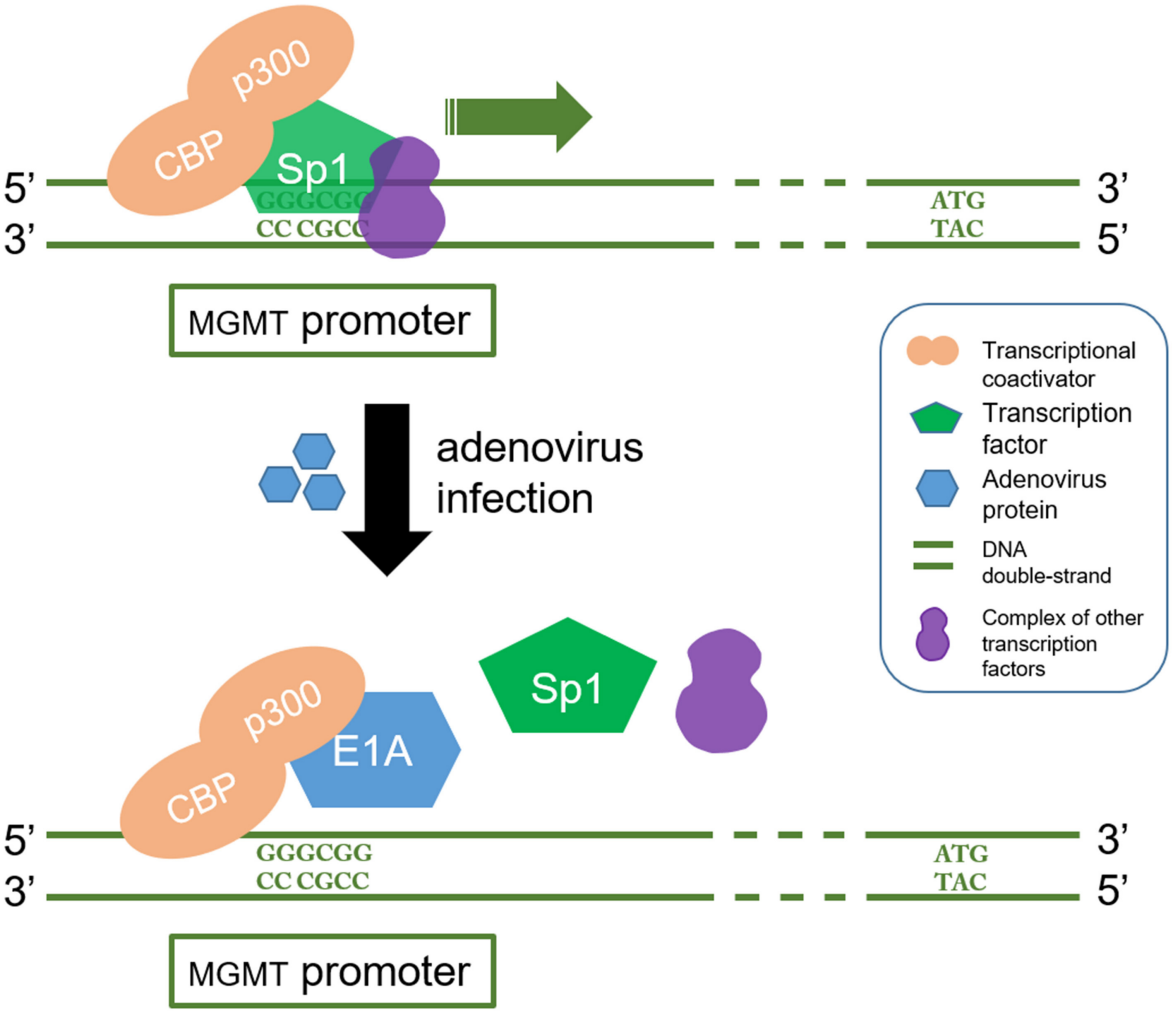
E1A interferes with CBP/p300 in which regulate the transcription process of MGMT gene. CBP/p300 is a transcription coactivator and Sp1 is a transcription factor. CBP/p300 recruits Sp1 protein to recognize and bind to the GC region (5'-GGGCGG-3') in the MGMT promoter, which in turn acts as a transcriptional activator. On the other hand, E1A is a product of oncolytic adenovirus. It stops the Sp1 from being recruited by binding to p300. This blocks the MGMT gene transcription signal.

Oncolytic viruses which inactivate or leverage the cellular DNA-repair machinery to achieve productive replication have also been exploited to design agents targeting MGMT. Adenoviruses express proteins which can downregulate MGMT expression. It has been reported that overexpression of adenovirus E1A, which binds p300, efficiently inhibits both basal and TSA inducible promoter activity of MGMT and may thus reduce chemoresistance (93, 94). CBP/p300 is a transcriptional coactivator which interacts with multiple transcription factors including those involved in MGMT gene. It plays an important role in many cellular processes, and the structural and functional versatility of CBP/p300 are yet to be fully elucidated. For this reason, the utilization of adenovirus targeting MGMT is far from being clinically implemented (95) (Figure 3).

Jiang et al. reported that a combination of TMZ and viral therapy may overcome the chemoresistance of gliomas to TMZ (96). Further, it has been shown that oncolytic virus-mediated manipulation of DNA damage responses can also be applied to kill GSCs (97). The discovery of this oncolytic viral therapy opens a new era in cancer therapy. However, there are safety concerns regarding the use of virus-based therapy.

Other drugs that target MGMT protein such as disulfiram (DSF) have also been studied. For instance, Paranjpe reported that DSF directly suppressed MGMT protein expression through sole site Cys145 (98).

## Conclusion and Perspectives

**Figure 4:**
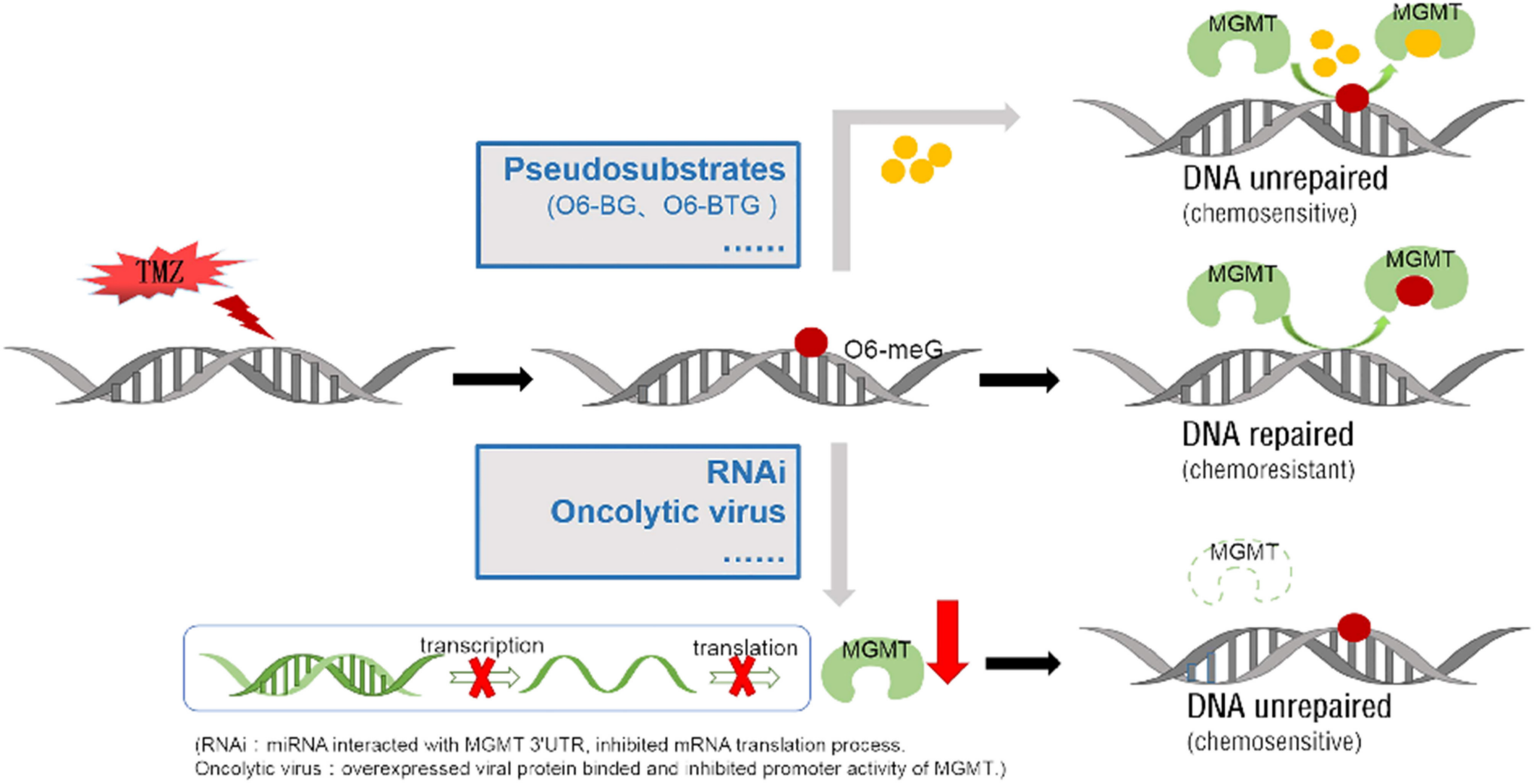
Two main approaches to block methylated DNA repair by targeting the MGMT protein. TMZ can add methyl to the guanine on the DNA molecule (form as 06-meG), which then induce DNA cross-linking. MGMT protein can repair DNA damage by binding and transferring the methyl on it. Low molecular weight O^6^-meG analogs, like O^6^-BG and O^6^-BTG, are used as pseudosubstrates to bind MGMT protein and reduce the methyl transfer activity. Specific miRNA/ liposome complexes which interact with MGMT 3'UTR then inhibit the MGMT mRNA translation process (RNAi). The proliferated oncolytic virus in a host can inhibit the host's MGMT promoter's activation, by means of the E1A binding to the p300.

MGMT is a DNA methyltransferase which repairs damaged DNA thus avoiding cell death caused by alkylating agents. The expression of MGMT gene is mainly regulated by epigenetic modification. Several methods have been developed for MGMT detection including immunohistochemistry, methylation-specific PCR, pyrophosphate sequencing, MGMT activity test, real-time quantitative PCR among others. Methylation of MGMT promoter can predict whether alkylating agents are effective for glioblastoma and low-grade gliomas. The prognostic value of MGMT methylation is still controversial and calls for further clarification. Epigenetic regulation of specific sites of MGMT CpG island influences MGMT transcription. Chemotherapy and radiotherapy may modulate MGMT methylation status, activity and protein expression. TMZ is a promising chemotherapeutic agent for glioma, but the rapid development of drug resistance poses a huge challenge. Overexpression of MGMT is an important mechanism of TMZ resistance. Several strategies have been pursued to improve the anti-tumor effects of TMZ. These include development of pseudosubstrates, RNAi, viral proteins and many others agents (Figure 4). Given on-going research advancements in this field, the current poor prognosis of glioma patients is expected to improve.

## Author Contributions

AS designed the study and determined the final version. QW revised the manuscript and polished the language. WY drafted the manuscript. LZ made the figures and tables. All authors read and approved the final manuscript.

### Conflict of Interest

The authors declare that the research was conducted in the absence of any commercial or financial relationships that could be construed as a potential conflict of interest.

## Abbreviations

MGMT: O^6^-methylguanine-DNA methyltransferase
MSP: methylation-specific PCR
IHC: immunohistochemistry
PCV: methylbenzylhydrazine, cyclohexanisolone and vincristine
OS: overall survival
PFS: progression-free survival
IDH1: isocitrate dehydrogenase
LMPCR: Linker-mediated PCR
TSS: transcription initiation site
BSP: Bisulfite Sequencing PCR
O^6^-BG: O^6^-benzylguanine
TMZ: temozolomide
CSF: cerebrospinal fluid
O^6^-BTG: O^6^-(4-bromothenyl) guanine
DSF: disulfiram.

## References

[1] Gardiner-Garden M Frommer M. CpG islands in vertebrate genomes. J Mol Biol. (1987) 196:261–82. 10.1016/0022-2836(87)90689-9 3656447

[2] Harris LC Potter PM Tano K Shiota S Mitra S Brent TP. Characterization of the promoter region of the human O6-methylguanine-DNA methyltransferase gene. Nucleic Acids Res. (1991) 19:6163–7. 10.1093/nar/19.22.6163 1956775 PMC329112

[3] Kent WJ. BLAT–the BLAST-like alignment tool. Genome Res. (2002) 12:656–64. 10.1101/gr.229202 11932250 PMC187518

[4] Pruitt KD Tatusova T Maglott DR. NCBI Reference Sequence (RefSeq): a curated non-redundant sequence database of genomes, transcripts and proteins. Nucleic Acids Res. (2005) 33:D501–4. 10.1093/nar/gki025 15608248 PMC539979

[5] Day RS III Ziolkowski CH Scudiero DA Meyer SA Lubiniecki AS Girardi AJ. Defective repair of alkylated DNA by human tumour and SV40-transformed human cell strains. Nature. (1980) 288:724–7. 10.1038/288724a0 6256643

[6] Fornace AJ Jr Papathanasiou MA Hollander MC Yarosh DB. Expression of the O6-methylguanine-DNA methyltransferase gene MGMT in MER+ and MER- human tumor cells. Cancer Res. (1990) 50:7908–11. 2253230

[7] Pieper RO Futscher BW Dong Q Ellis TM Erickson LC. Comparison of O-6-methylguanine DNA methyltransferase (MGMT) mRNA levels in Mer+ and Mer- human tumor cell lines containing the MGMT gene by the polymerase chain reaction technique. Cancer Commun. (1990) 2:13–20. 10.3727/095535490820874812 2369549

[8] Kroes RA Erickson LC. The role of mRNA stability and transcription in O6-methylguanine DNA methyltransferase (MGMT) expression in Mer+ human tumor cells. Carcinogenesis. (1995) 16:2255–7. 10.1093/carcin/16.9.2255 7554086

[9] Bouras E Karakioulaki M Bougioukas KI Aivaliotis M Tzimagiorgis G Chourdakis M. Gene promoter methylation and cancer: an umbrella review. Gene. (2019) 710:333–40. 10.1016/j.gene.2019.06.023 31202904

[10] Costello JF Futscher BW Tano K Graunke DM Pieper RO. Graded methylation in the promoter and body of the O6-methylguanine DNA methyltransferase (MGMT) gene correlates with MGMT expression in human glioma cells. J Biol Chem. (1994) 269:17228–37. 8006031

[11] Watts GS Pieper RO Costello JF Peng YM Dalton WS Futscher BW. Methylation of discrete regions of the O6-methylguanine DNA methyltransferase (MGMT) CpG island is associated with heterochromatinization of the MGMT transcription start site and silencing of the gene. Mol Cell Biol. (1997) 17:5612–9. 10.1128/MCB.17.9.5612 9271436 PMC232409

[12] Costello JF Futscher BW Kroes RA Pieper RO. Methylation-related chromatin structure is associated with exclusion of transcription factors from and suppressed expression of the O-6-methylguanine DNA methyltransferase gene in human glioma cell lines. Mol Cell Biol. (1994) 14:6515–21. 10.1128/MCB.14.10.6515 7523853 PMC359181

[13] Micko ASG Hoftberger R Wohrer A Millesi M Knosp E Wolfsberger S. MGMT assessment in pituitary adenomas: comparison of different immunohistochemistry fixation chemicals. Pituitary. (2018) 21:266–73. 10.1007/s11102-018-0862-x 29344904 PMC5942339

[14] Jiang X Reardon DA Desjardins A Vredenburgh JJ Quinn JA Austin AD. O6-methylguanine-DNA methyltransferase (MGMT) immunohistochemistry as a predictor of resistance to temozolomide in primary CNS lymphoma. J Neurooncol. (2013) 114:135–40. 10.1007/s11060-013-1162-y 23686298

[15] Mason S McDonald K. MGMT testing for glioma in clinical laboratories: discordance with methylation analyses prevents the implementation of routine immunohistochemistry. J Cancer Res Clin Oncol. (2012) 138:1789–97. 10.1007/s00432-012-1312-1 22986811 PMC11824796

[16] Preusser M Charles Janzer R Felsberg J Reifenberger G Hamou MF Diserens AC. Anti-O6-methylguanine-methyltransferase (MGMT) immunohistochemistry in glioblastoma multiforme: observer variability and lack of association with patient survival impede its use as clinical biomarker. Brain Pathol. (2008) 18:520–32. 10.1111/j.1750-3639.2008.00153.x 18400046 PMC8095504

[17] Christmann M Nagel G Horn S Krahn U Wiewrodt D Sommer C. MGMT activity, promoter methylation and immunohistochemistry of pretreatment and recurrent malignant gliomas: a comparative study on astrocytoma and glioblastoma. Int J Cancer. (2010) 127:2106–18. 10.1002/ijc.25229 20131314

[18] Yachi K Watanabe T Ohta T Fukushima T Yoshino A Ogino A. Relevance of MSP assay for the detection of MGMT promoter hypermethylation in glioblastomas. Int J Oncol. (2008) 33:469–75. 10.3892/ijo_00000029 18695875

[19] Herman JG Graff JR Myohanen S Nelkin BD Baylin SB. Methylation-specific PCR: a novel PCR assay for methylation status of CpG islands. Proc Natl Acad Sci USA. (1996) 93:9821–6. 10.1073/pnas.93.18.9821 8790415 PMC38513

[20] Switzeny OJ Christmann M Renovanz M Giese A Sommer C Kaina B. MGMT promoter methylation determined by HRM in comparison to MSP and pyrosequencing for predicting high-grade glioma response. Clin Epigenetics. (2016) 8:49. 10.1186/s13148-016-0204-7 27158275 PMC4858829

[21] Tost J El abdalaoui H Gut IG. Serial pyrosequencing for quantitative DNA methylation analysis. Biotechniques. (2006) 40:721–2. 10.2144/000112190 16774114

[22] Schnell O Albrecht V Pfirrmann D Eigenbrod S Krebs B Romagna A MGMT promoter methylation is not correlated with integrin expression in malignant gliomas: clarifying recent clinical trial results. Med Oncol. (2018) 35:103 10.1007/s12032-018-1162-z 29882028

[23] Toffolatti L Scquizzato E Cavallin S Canal F Scarpa M Stefani PM. MGMT promoter methylation and correlation with protein expression in primary central nervous system lymphoma. Virchows Arch. (2014) 465:579–86. 10.1007/s00428-014-1622-6 25031012

[24] Melguizo C Prados J Gonzalez B Ortiz R Concha A Alvarez PJ. MGMT promoter methylation status and MGMT and CD133 immunohistochemical expression as prognostic markers in glioblastoma patients treated with temozolomide plus radiotherapy. J Transl Med. (2012) 10:250. 10.1186/1479-5876-10-250 23245659 PMC3551841

[25] Uno M Oba-Shinjo SM Camargo AA Moura RP Aguiar PH Cabrera HN. Correlation of MGMT promoter methylation status with gene and protein expression levels in glioblastoma. Clinics. (2011) 66:1747–55. 10.1590/S1807-59322011001000013 22012047 PMC3180167

[26] Wang L Li Z Liu C Chen L Liu L Hu Z. Comparative assessment of three methods to analyze MGMT methylation status in a series of 350 gliomas and gangliogliomas. Pathol Res Pract. (2017) 213:1489–93. 10.1016/j.prp.2017.10.007 29103769

[27] Kaina B Christmann M. DNA repair in personalized brain cancer therapy with temozolomide and nitrosoureas. DNA Repair. (2019) 78:128–41. 10.1016/j.dnarep.2019.04.007 31039537

[28] Criniere E Kaloshi G Laigle-Donadey F Lejeune J Auger N Benouaich-Amiel A. MGMT prognostic impact on glioblastoma is dependent on therapeutic modalities. J Neurooncol. (2007) 83:173–9. 10.1007/s11060-006-9320-0 17219056

[29] Hegi ME Diserens AC Gorlia T Hamou MF de Tribolet N Weller M. MGMT gene silencing and benefit from temozolomide in glioblastoma. N Engl J Med. (2005) 352:997–1003. 10.1056/NEJMoa043331 15758010

[30] Reifenberger G Hentschel B Felsberg J Schackert G Simon M Schnell O. Predictive impact of MGMT promoter methylation in glioblastoma of the elderly. Int J Cancer. (2012) 131:1342–50. 10.1002/ijc.27385 22139906

[31] Esteller M Garcia-Foncillas J Andion E Goodman SN Hidalgo OF Vanaclocha V. Inactivation of the DNA-repair gene MGMT and the clinical response of gliomas to alkylating agents. N Engl J Med. (2000) 343:1350–4. 10.1056/NEJM200011093431901 11070098

[32] Hegi ME Diserens AC Godard S Dietrich PY Regli L Ostermann S. Clinical trial substantiates the predictive value of O-6-methylguanine-DNA methyltransferase promoter methylation in glioblastoma patients treated with temozolomide. Clin Cancer Res. (2004) 10:1871–4. 10.1158/1078-0432.CCR-03-0384 15041700

[33] Everhard S Kaloshi G Criniere E Benouaich-Amiel A Lejeune J Marie Y. MGMT methylation: a marker of response to temozolomide in low-grade gliomas. Ann Neurol. (2006) 60:740–3. 10.1002/ana.21044 17192931

[34] Aoki K Natsume A. Overview of DNA methylation in adult diffuse gliomas. Brain Tumor Pathol. (2019) 36:84–91. 10.1007/s10014-019-00339-w 30937703

[35] Qi F Yin Z Wang G Zeng S Clinical and prognostic significance of O(6)-methylguanine-DNA methyltransferase promoter methylation in patients with melanoma: a systematic meta-analysis. Ann Dermatol. (2018) 30:129–35. 10.5021/ad.2018.30.2.129 29606808 PMC5839882

[36] Dahlrot RH Larsen P Boldt HB Kreutzfeldt MS Hansen S Hjelmborg JB. Posttreatment effect of MGMT methylation level on glioblastoma survival. J Neuropathol Exp Neurol. (2019) 78:633–40. 10.1093/jnen/nlz032 31058280 PMC6581556

[37] Pandith AA Qasim I Zahoor W Shah P Bhat AR Sanadhya D. Concordant association validates MGMT methylation and protein expression as favorable prognostic factors in glioma patients on alkylating chemotherapy (Temozolomide). Sci Rep. (2018) 8:6704. 10.1038/s41598-018-25169-2 29712977 PMC5928198

[38] Malmstrom A Gronberg BH Marosi C Stupp R Frappaz D Schultz H. Temozolomide versus standard 6-week radiotherapy versus hypofractionated radiotherapy in patients older than 60 years with glioblastoma: the Nordic randomised, phase 3 trial. Lancet Oncol. (2012) 13:916–26. 10.1016/S1470-2045(12)70265-6 22877848

[39] Wick W Platten M Meisner C Felsberg J Tabatabai G Simon M. Temozolomide chemotherapy alone versus radiotherapy alone for malignant astrocytoma in the elderly: the NOA-08 randomised, phase 3 trial. Lancet Oncol. (2012) 13:707–15. 10.1016/S1470-2045(12)70164-X 22578793

[40] van den Bent MJ Dubbink HJ Sanson M van der Lee-Haarloo CR Hegi M Jeuken JW MGMT promoter methylation is prognostic but not predictive for outcome to adjuvant PCV chemotherapy in anaplastic oligodendroglial tumors: a report from EORTC Brain Tumor Group Study 26951. J Clin Oncol. (2009) 27:5881–6. 10.1200/JCO.2009.24.1034 19901104 PMC2793037

[41] Wick W Hartmann C Engel C Stoffels M Felsberg J Stockhammer F NOA-04 randomized phase III trial of sequential radiochemotherapy of anaplastic glioma with procarbazine, lomustine, and vincristine or temozolomide. J Clin Oncol. (2009) 27:5874–80. 10.1200/JCO.2009.23.6497 19901110

[42] Gilbert MR Wang M Aldape KD Stupp R Hegi M Jaeckle KA RTOG 0525: a randomized phase III trial comparing standard adjuvant temozolomide (TMZ) with a dose-dense (dd) schedule in newly diagnosed glioblastoma (GBM). J Clin Oncol. (2011) 29:2006 10.1200/jco.2011.29.15_suppl.2006

[43] Pieper RO Patel S Ting SA Futscher BW Costello JF. Methylation of CpG island transcription factor binding sites is unnecessary for aberrant silencing of the human MGMT gene. J Biol Chem. (1996) 271:13916–24. 10.1074/jbc.271.23.13916 8662860

[44] Qian XC Brent TP. Methylation hot spots in the 5' flanking region denote silencing of the O6-methylguanine-DNA methyltransferase gene. Cancer Res. (1997) 57:3672–7. 9288770

[45] Malley DS Hamoudi RA Kocialkowski S Pearson DM Collins VP Ichimura K. A distinct region of the MGMT CpG island critical for transcriptional regulation is preferentially methylated in glioblastoma cells and xenografts. Acta Neuropathol. (2011) 121:651–61. 10.1007/s00401-011-0803-5 21287394

[46] Everhard S Tost J El Abdalaoui H Criniere E Busato F Marie Y. Identification of regions correlating MGMT promoter methylation and gene expression in glioblastomas. Neuro Oncol. (2009) 11:348–56. 10.1215/15228517-2009-001 19224763 PMC2743215

[47] Bady P Sciuscio D Diserens AC Bloch J van den Bent MJ Marosi C. MGMT methylation analysis of glioblastoma on the Infinium methylation BeadChip identifies two distinct CpG regions associated with gene silencing and outcome, yielding a prediction model for comparisons across datasets, tumor grades, and CIMP-status. Acta Neuropathol. (2012) 124:547–60. 10.1007/s00401-012-1016-2 22810491 PMC3444709

[48] Mur P Rodriguez de Lope A Diaz-Crespo FJ Hernandez-Iglesias T Ribalta T Fiano C. Impact on prognosis of the regional distribution of MGMT methylation with respect to the CpG island methylator phenotype and age in glioma patients. J Neurooncol. (2015) 122:441–50. 10.1007/s11060-015-1738-9 25682093

[49] Wiewrodt D Nagel G Dreimuller N Hundsberger T Perneczky A Kaina B. MGMT in primary and recurrent human glioblastomas after radiation and chemotherapy and comparison with p53 status and clinical outcome. Int J Cancer. (2008) 122:1391–9. 10.1002/ijc.23219 18000822

[50] Brandes AA Franceschi E Tosoni A Bartolini S Bacci A Agati R. O(6)-methylguanine DNA-methyltransferase methylation status can change between first surgery for newly diagnosed glioblastoma and second surgery for recurrence: clinical implications. Neuro Oncol. (2010) 12:283–8. 10.1093/neuonc/nop050 20167816 PMC2940594

[51] Felsberg J Thon N Eigenbrod S Hentschel B Sabel MC Westphal M. Promoter methylation and expression of MGMT and the DNA mismatch repair genes MLH1, MSH2, MSH6 and PMS2 in paired primary and recurrent glioblastomas. Int J Cancer. (2011) 129:659–70. 10.1002/ijc.26083 21425258

[52] Fritz G Tano K Mitra S Kaina B. Inducibility of the DNA repair gene encoding O6-methylguanine-DNA methyltransferase in mammalian cells by DNA-damaging treatments. Mol Cell Biol. (1991) 11:4660–8. 10.1128/MCB.11.9.4660 1875945 PMC361355

[53] Chan CL Wu Z Eastman A Bresnick E. Irradiation-induced expression of O6-methylguanine-DNA methyltransferase in mammalian cells. Cancer Res. (1992) 52:1804–9. 1372530

[54] Grombacher T Mitra S Kaina B. Induction of the alkyltransferase (MGMT) gene by DNA damaging agents and the glucocorticoid dexamethasone and comparison with the response of base excision repair genes. Carcinogenesis. (1996) 17:2329–36. 10.1093/carcin/17.11.2329 8968045

[55] Boldogh I Ramana CV Chen Z Biswas T Hazra TK Grosch S. Regulation of expression of the DNA repair gene O6-methylguanine-DNA methyltransferase via protein kinase C-mediated signaling. Cancer Res. (1998) 58:3950–6. 9731508

[56] Aasland D Reich TR Tomicic MT Switzeny OJ Kaina B Christmann M Repair gene O(6) -methylguanine-DNA methyltransferase is controlled by SP1 and up-regulated by glucocorticoids, but not by temozolomide and radiation. J Neurochem. (2018) 144:139–51. 10.1111/jnc.14262 29164620

[57] Tomicic MT Meise R Aasland D Berte N Kitzinger R Kramer OH. Apoptosis induced by temozolomide and nimustine in glioblastoma cells is supported by JNK/c-Jun-mediated induction of the BH3-only protein BIM. Oncotarget. (2015) 6:33755–68. 10.18632/oncotarget.5274 26418950 PMC4741800

[58] Pitter KL Tamagno I Alikhanyan K Hosni-Ahmed A Pattwell SS Donnola S. Corticosteroids compromise survival in glioblastoma. Brain. (2016) 139(Pt 5):1458–71. 10.1093/brain/aww046 27020328 PMC5006251

[59] Pistollato F Abbadi S Rampazzo E Persano L Della Puppa A Frasson C. Intratumoral hypoxic gradient drives stem cells distribution and MGMT expression in glioblastoma. Stem Cells. (2010) 28:851–62. 10.1002/stem.415 20309962

[60] Liu G Yuan X Zeng Z Tunici P Ng H Abdulkadir IR. Analysis of gene expression and chemoresistance of CD133+ cancer stem cells in glioblastoma. Mol Cancer. (2006) 5:67. 10.1186/1476-4598-5-67 17140455 PMC1697823

[61] Beier D Hau P Proescholdt M Lohmeier A Wischhusen J Oefner PJ. CD133(+) and CD133(-) glioblastoma-derived cancer stem cells show differential growth characteristics and molecular profiles. Cancer Res. (2007) 67:4010–5. 10.1158/0008-5472.CAN-06-4180 17483311

[62] Beier D Rohrl S Pillai DR Schwarz S Kunz-Schughart LA Leukel P. Temozolomide preferentially depletes cancer stem cells in glioblastoma. Cancer Res. (2008) 68:5706–15. 10.1158/0008-5472.CAN-07-6878 18632623

[63] Mantwill K Naumann U Seznec J Girbinger V Lage H Surowiak P. YB-1 dependent oncolytic adenovirus efficiently inhibits tumor growth of glioma cancer stem like cells. J Transl Med. (2013) 11:216. 10.1186/1479-5876-11-216 24044901 PMC3848904

[64] Happold C Stojcheva N Silginer M Weiss T Roth P Reifenberger G Transcriptional control of O(6) -methylguanine DNA methyltransferase expression and temozolomide resistance in glioblastoma. J Neurochem. (2018) 144:780–90. 10.1111/jnc.14326 29480969

[65] Chumakova A Lathia JD Outlining involvement of stem cell program in regulation of O6-methylguanine DNA methyltransferase and development of temozolomide resistance in glioblastoma: an editorial highlight for 'Transcriptional control of O(6) -methylguanine DNA methyltransferase expression and temozolomide resistance in glioblastoma' on page 780. J Neurochem. (2018) 144:688–90. 10.1111/jnc.14280 29644711

[66] Middleton MR Margison GP. Improvement of chemotherapy efficacy by inactivation of a DNA-repair pathway. Lancet Oncol. (2003) 4:37–44. 10.1016/S1470-2045(03)00959-8 12517538

[67] Bobola MS Silber JR Ellenbogen RG Geyer JR Blank A Goff RD. O6-methylguanine-DNA methyltransferase, O^6^-benzylguanine, and resistance to clinical alkylators in pediatric primary brain tumor cell lines. Clin Cancer Res. (2005) 11:2747–55. 10.1158/1078-0432.CCR-04-2045 15814657

[68] Koch D Hundsberger T Boor S Kaina B. Local intracerebral administration of O(6)-benzylguanine combined with systemic chemotherapy with temozolomide of a patient suffering from a recurrent glioblastoma. J Neurooncol. (2007) 82:85–9. 10.1007/s11060-006-9244-8 17031555

[69] Quinn JA Desjardins A Weingart J Brem H Dolan ME Delaney SM Phase I trial of temozolomide plus O^6^-benzylguanine for patients with recurrent or progressive malignant glioma. J Clin Oncol. (2005) 23:7178–87. 10.1200/JCO.2005.06.502 16192602

[70] Quinn JA Jiang SX Reardon DA Desjardins A Vredenburgh JJ Rich JN. Phase I trial of temozolomide plus O^6^-benzylguanine 5-day regimen with recurrent malignant glioma. Neuro Oncol. (2009) 11:556–61. 10.1215/15228517-2009-007 19289491 PMC2765345

[71] Verbeek B Southgate TD Gilham DE Margison GP. O6-methylguanine-DNA methyltransferase inactivation and chemotherapy. Br Med Bull. (2008) 85:17–33. 10.1093/bmb/ldm036 18245773

[72] Broniscer A Gururangan S MacDonald TJ Goldman S Packer RJ Stewart CF. Phase I trial of single-dose temozolomide and continuous administration of O^6^-benzylguanine in children with brain tumors: a pediatric brain tumor consortium report. Clin Cancer Res. (2007) 13(22 Pt 1):6712–8. 10.1158/1078-0432.CCR-07-1016 18006772

[73] Quinn JA Jiang SX Carter J Reardon DA Desjardins A Vredenburgh JJ. Phase II trial of gliadel plus O^6^-benzylguanine in adults with recurrent glioblastoma multiforme. Clin Cancer Res. (2009) 15:1064–8. 10.1158/1078-0432.CCR-08-2130 19188181 PMC2710963

[74] Barvaux VA Lorigan P Ranson M Gillum AM McElhinney RS McMurry TB. Sensitization of a human ovarian cancer cell line to temozolomide by simultaneous attenuation of the Bcl-2 antiapoptotic protein and DNA repair by O6-alkylguanine-DNA alkyltransferase. Mol Cancer Ther. (2004) 3:1215–20. 15486188

[75] Turriziani M Caporaso P Bonmassar L Buccisano F Amadori S Venditti A. O6-(4-bromothenyl)guanine (PaTrin-2), a novel inhibitor of O6-alkylguanine DNA alkyl-transferase, increases the inhibitory activity of temozolomide against human acute leukaemia cells *in vitro*. Pharmacol Res. (2006) 53:317–23. 10.1016/j.phrs.2005.12.001 16412662

[76] Middleton MR Kelly J Thatcher N Donnelly DJ McElhinney RS McMurry TB. O(6)-(4-bromothenyl)guanine improves the therapeutic index of temozolomide against A375M melanoma xenografts. Int J Cancer. (2000) 85:248–52. 10.1002/(SICI)1097-0215(20000115)85:2<248::AID-IJC16>3.0.CO;2-V 10629085

[77] Clemons M Kelly J Watson AJ Howell A McElhinney RS McMurry TB. O6-(4-bromothenyl)guanine reverses temozolomide resistance in human breast tumour MCF-7 cells and xenografts. Br J Cancer. (2005) 93:1152–6. 10.1038/sj.bjc.6602833 16278661 PMC2361498

[78] Ranson M Middleton MR Bridgewater J Lee SM Dawson M Jowle D. Lomeguatrib, a potent inhibitor of O6-alkylguanine-DNA-alkyltransferase: phase I safety, pharmacodynamic, and pharmacokinetic trial and evaluation in combination with temozolomide in patients with advanced solid tumors. Clin Cancer Res. (2006) 12:1577–84. 10.1158/1078-0432.CCR-05-2198 16533784

[79] Papachristodoulou A Signorell RD Werner B Brambilla D Luciani P Cavusoglu M. Chemotherapy sensitization of glioblastoma by focused ultrasound-mediated delivery of therapeutic liposomes. J Control Release. (2019) 295:130–9. 10.1016/j.jconrel.2018.12.009 30537486

[80] Reinhard J Eichhorn U Wiessler M Kaina B. Inactivation of O(6)-methylguanine-DNA methyltransferase by glucose-conjugated inhibitors. Int J Cancer. (2001) 93:373–9. 10.1002/ijc.1336 11433402

[81] Kaina B Muhlhausen U Piee-Staffa A Christmann M Garcia Boy R Rosch F. Inhibition of O6-methylguanine-DNA methyltransferase by glucose-conjugated inhibitors: comparison with nonconjugated inhibitors and effect on fotemustine and temozolomide-induced cell death. J Pharmacol Exp Ther. (2004) 311:585–93. 10.1124/jpet.104.071316 15254145

[82] Tomaszowski KH Schirrmacher R Kaina B. Multidrug efflux pumps attenuate the effect of MGMT inhibitors. Mol Pharm. (2015) 12:3924–34. 10.1021/acs.molpharmaceut.5b00341 26379107

[83] Tomaszowski KH Hellmann N Ponath V Takatsu H Shin HW Kaina B. Uptake of glucose-conjugated MGMT inhibitors in cancer cells: role of flippases and type IV P-type ATPases. Sci Rep. (2017) 7:13925. 10.1038/s41598-017-14129-x 29066805 PMC5655675

[84] Javanmard S Loktionova NA Fang Q Pauly GT Pegg AE Moschel RC. Inactivation of O(6)-alkylguanine-DNA alkyltransferase by folate esters of O(6)-benzyl-2'-deoxyguanosine and of O(6)-[4-(hydroxymethyl)benzyl]guanine. J Med Chem. (2007) 50:5193–201. 10.1021/jm0705859 17880193 PMC2597536

[85] Watson AJ Middleton MR McGown G Thorncroft M Ranson M Hersey P O(6)-methylguanine-DNA methyltransferase depletion and DNA damage in patients with melanoma treated with temozolomide alone or with lomeguatrib. Br J Cancer. (2009) 100:1250–6. 10.1038/sj.bjc.6605015 19367283 PMC2676560

[86] Watson AJ Sabharwal A Thorncroft M McGown G Kerr R Bojanic S. Tumor O(6)-methylguanine-DNA methyltransferase inactivation by oral lomeguatrib. Clin Cancer Res. (2010) 16:743–9. 10.1158/1078-0432.CCR-09-1389 20068091 PMC2807621

[87] Quinn JA Jiang SX Reardon DA Desjardins A Vredenburgh JJ Rich JN. Phase II trial of temozolomide plus O^6^-benzylguanine in adults with recurrent, temozolomide-resistant malignant glioma. J Clin Oncol. (2009) 27:1262–7. 10.1200/JCO.2008.18.8417 19204199 PMC2667825

[88] Tawbi HA Villaruz L Tarhini A Moschos S Sulecki M Viverette F. Inhibition of DNA repair with MGMT pseudosubstrates: phase I study of lomeguatrib in combination with dacarbazine in patients with advanced melanoma and other solid tumours. Br J Cancer. (2011) 105:773–7. 10.1038/bjc.2011.285 21811257 PMC3171007

[89] Romani M Pistillo MP Banelli B. Epigenetic targeting of glioblastoma. Front Oncol. (2018) 8:448. 10.3389/fonc.2018.00448 30386738 PMC6198064

[90] Kato T Natsume A Toda H Iwamizu H Sugita T Hachisu R. Efficient delivery of liposome-mediated MGMT-siRNA reinforces the cytotoxity of temozolomide in GBM-initiating cells. Gene Ther. (2010) 17:1363–71. 10.1038/gt.2010.88 20520650

[91] Zhang W Zhang J Hoadley K Kushwaha D Ramakrishnan V Li S. miR-181d: a predictive glioblastoma biomarker that downregulates MGMT expression. Neuro Oncol. (2012) 14:712–9. 10.1093/neuonc/nos089 22570426 PMC3367855

[92] Nie E Jin X Wu W Yu T Zhou X Shi Z. MiR-198 enhances temozolomide sensitivity in glioblastoma by targeting MGMT. J Neurooncol. (2017) 133:59–68. 10.1007/s11060-017-2425-9 28425046

[93] Bhakat KK Mitra S. Regulation of the human O(6)-methylguanine-DNA methyltransferase gene by transcriptional coactivators cAMP response element-binding protein-binding protein and p300. J Biol Chem. (2000) 275:34197–204. 10.1074/jbc.M005447200 10942771

[94] Alonso MM Gomez-Manzano C Bekele BN Yung WK Fueyo J. Adenovirus-based strategies overcome temozolomide resistance by silencing the O6-methylguanine-DNA methyltransferase promoter. Cancer Res. (2007) 67:11499–504. 10.1158/0008-5472.CAN-07-5312 18089777

[95] Wang F Marshall CB Ikura M. Transcriptional/epigenetic regulator CBP/p300 in tumorigenesis: structural and functional versatility in target recognition. Cell Mol Life Sci. (2013) 70:3989–4008. 10.1007/s00018-012-1254-4 23307074 PMC11113169

[96] Jiang H Alonso MM Gomez-Manzano C Piao Y Fueyo J. Oncolytic viruses and DNA-repair machinery: overcoming chemoresistance of gliomas. Expert Rev Anticancer Ther. (2006) 6:1585–92. 10.1586/14737140.6.11.1585 17134363

[97] Kanai R Rabkin SD Yip S Sgubin D Zaupa CM Hirose Y. Oncolytic virus-mediated manipulation of DNA damage responses: synergy with chemotherapy in killing glioblastoma stem cells. JNCI J Natl Cancer Inst. (2012) 104:42–55. 10.1093/jnci/djr509 22173583 PMC3250384

[98] Paranjpe A Zhang R Ali-Osman F Bobustuc GC Srivenugopal KS. Disulfiram is a direct and potent inhibitor of human O6-methylguanine-DNA methyltransferase (MGMT) in brain tumor cells and mouse brain and markedly increases the alkylating DNA damage. Carcinogenesis. (2014) 35:692–702. 10.1093/carcin/bgt366 24193513 PMC3941740

